# Menthol and Other Flavor Chemicals in Cigarettes from Vietnam and the Philippines

**DOI:** 10.1093/ntr/ntad146

**Published:** 2023-08-14

**Authors:** Joanna E Cohen, Lauren Czaplicki, Elizabeth Crespi, Jennifer L Brown, Wentai Luo, Kevin J McWhirter, Braden C Masanga, James F Pankow

**Affiliations:** Institute for Global Tobacco Control (IGTC), Department of Health, Behavior and Society, Johns Hopkins Bloomberg School of Public Health, Baltimore, MD 21205, USA; Institute for Global Tobacco Control (IGTC), Department of Health, Behavior and Society, Johns Hopkins Bloomberg School of Public Health, Baltimore, MD 21205, USA; Institute for Global Tobacco Control (IGTC), Department of Health, Behavior and Society, Johns Hopkins Bloomberg School of Public Health, Baltimore, MD 21205, USA; Institute for Global Tobacco Control (IGTC), Department of Health, Behavior and Society, Johns Hopkins Bloomberg School of Public Health, Baltimore, MD 21205, USA; Department of Civil and Environmental Engineering, Portland State University, Portland, Oregon 97207, USA; Department of Civil and Environmental Engineering, Portland State University, Portland, Oregon 97207, USA; Department of Civil and Environmental Engineering, Portland State University, Portland, Oregon 97207, USA; Department of Civil and Environmental Engineering, Portland State University, Portland, Oregon 97207, USA

## Abstract

**Introduction:**

Tobacco product flavors can increase product appeal, adolescent initiation and experimentation, and difficulty quitting. Flavored tobacco products are not restricted in Vietnam or the Philippines despite the high smoking prevalence among those 15 years of age and older (24% and 23%, respectively). There are no published reports to our knowledge on the levels of flavor chemicals in the cigarettes sold in these two countries.

**Methods:**

Cigarettes were purchased in Vietnam (32 brand variants) and the Philippines (19 brand variants) during 2020. Chemical analyses gave the mg/filter, mg/rod, and mg/stick (= mg/(filter + rod)) values for 180 individual flavor chemicals. Values were calculated for menthol, clove-related compounds, and “other flavor chemicals” (OFCs).

**Results:**

Five flavor groupings were found among the brand variants purchased in Vietnam: menthol + OFCs (*n* = 15), OFCs only (*n* = 8), nonflavored (*n* = 7), menthol + OFCs with a clove flavorant (*n* = 1) and menthol only (*n* = 1). Three flavor groupings were found among the brand variants purchased in the Philippines: menthol + OFCs (*n* = 10), nonflavored (*n* = 5), and menthol only (*n* = 4).

**Conclusions:**

A range of flavored cigarette products are being offered by tobacco companies in Vietnam and the Philippines, presumably to maximize cigarette sales. Regulation of flavor chemicals should be considered in these two countries.

**Implications:**

Article 9 of the WHO Framework Convention on Tobacco Control (FCTC), ratified by both Vietnam and the Philippines, states that “there is no justification for permitting the use of ingredients, such as flavoring agents, which help make tobacco products attractive.” Flavors increase product appeal, adolescent initiation and experimentation, and difficulty quitting. These analyses found that cigarettes purchased in Vietnam and the Philippines contained menthol and other flavor chemicals. Tobacco companies are offering multiple flavor chemical profiles and nominally nonflavored versions in these countries; regulation of flavor chemicals should be considered in these two countries.

## Introduction

The Framework Convention on Tobacco Control (FCTC) of the World Health Organization requires that parties implement interventions to address the causes of the globalized tobacco epidemic. As of September 2022, 182 countries—including Vietnam and the Philippines—have signed and ratified the convention, committing to implement steps to reduce tobacco supply and demand. A key FCTC aim is to reduce the appeal of tobacco products^1^: Article 9 states “From the perspective of public health, there is no justification for permitting the use of ingredients, such as flavoring agents, which help make tobacco products attractive.”^2^ Flavors can increase product appeal by masking the undesirable taste or sensation of tobacco use^3^; the availability of flavored tobacco contributes to tobacco product initiation and experimentation among adolescents and difficulty quitting tobacco among those who regularly use flavored tobacco.^4^ Capsules have become a common vehicle for manufacturers to provide user-controlled release of flavor chemicals in cigarettes; the user crushes the capsule which is often a small sphere containing gelatin and flavoring liquid placed in the filter.^5–7^ Flavor threads are another approach to alter smoke taste and cigarette appearance; they are inserted into the filter and can be colored. Overall, there is insufficient data on flavored and menthol tobacco products, particularly in low- and middle-income countries, which might hinder the development of effective regulation to reduce the appeal of tobacco products.^8^

Although Vietnam and the Philippines are parties to the FCTC, flavored cigarettes are not regulated in either country. One-quarter of adults in Vietnam (24%) and the Philippines (23%) smoke combustible tobacco, and around 10% of all people who smoke in the Western Pacific region live in Vietnam or the Philippines.^9^ Current estimates of flavored tobacco use in Vietnam or the Philippines are limited. The 2015 Philippines Global Adult Tobacco Survey found over half (52%) of people who smoke manufactured cigarettes last purchased menthol cigarettes.^10^ In addition, data from our Tobacco Pack Surveillance System (TPackSS) study suggests a substantial proportion of cigarette packs sold in Vietnam and the Philippines in 2015 and 2016, were flavored based on content coding of flavor-related descriptions or imagery on the cigarette pack.^11^ While exact market share estimates vary, the Philippines has one of the largest market shares of menthol cigarettes^12,13^ and the flavored cigarette market in Vietnam is growing.^14,15^ Knowledge about the chemical composition of cigarettes can inform government regulation of cigarette ingredients and additives. In this study, we determine and compare the identities and levels of 180 flavor chemicals present in cigarettes sold in Vietnam and the Philippines during 2020. Results can inform flavor-related policy initiatives.

## Methods

We purchased a convenience sample of 51 cigarette packs, chosen to include products expected to be flavored and unflavored based on their brand variant names or packaging. In Vietnam, 32 unique cigarette packs were purchased in Hanoi in August 2020 from convenience stores, tobacco shops and online sites accessible in Vietnam. Three of the packs contained cigarillos (henceforth referred to as “cigarettes” for simplicity) and one pack was a “sampler” pack which contained four different color-coded versions, giving a total of 31 + 4 = 35 cigarette variants. In the Philippines, 19 unique cigarette packs were purchased in Manila in September 2020, predominantly from convenience stores and tobacco shops.

Upon purchase, each pack was put into a “barrier foil” Ziplock pouch (Ted Pella, Inc., Redding, CA) and sent by courier to Portland State University for analysis. Upon receipt, the samples were stored at 4 °C until analyzed. Each of the products was extracted and analyzed within two weeks of receipt.

### Analytical Methods

We analyzed all samples for 180 flavor chemicals. We utilized the solvent extraction method (using isopropanol) and instrumental analysis methods (using gas chromatography/mass spectrometry (GC/MS)—a method to identify different substances within a test sample) as described in prior studies of cigarettes purchased in Mexico,^16^ and kreteks (clove + tobacco cigarettes) and cigarettes purchased in Indonesia.^17^ Briefly, for each analysis of a brand variant, two sticks were separated into the filter and rod sections (the rod is the part of the cigarette that does not include the filter). The filters (with flavor thread or capsule(s)) were combined, the capsule(s) if present were crushed, the surrogate standard compound mass (0.8 mg) was added, and the isopropanol extraction solvent was added. The rod sections were similarly combined, the surrogate standard compound mass (0.8 mg) was added, and the isopropanol extraction solvent was added. Analysis in duplicate for the filters and for the rods accordingly required four sticks.

### Surrogate Standard (SS) Recoveries

The surrogate standard (SS) compound used in all analyses to monitor extraction recovery was 1,3,5-trichlorobenzene, added at 0.8 mg to each sample prior to the solvent extraction step. Determination of the SS amount found at the end of the analysis (along with the target analyte amounts) allowed calculation of the SS extraction recovery, in percent, the ideal value being 100.0%.

For all of the cigarette filter and rod samples, the calculated SS extraction recoveries ranged from 71.0 to 101.6%; the average recovery was 94.0% (± 5.6% standard deviation (sd)). Some black particles were visible in the filters of five of the Vietnam variants and in the filters of three of the Philippines variants. The tobacco industry has long used activated carbon in cigarette filters as an agent to capture smoke constituents that impair taste.^18^ There were three filter samples for which the SS recoveries were less than 80%; all contained carbon and were bought in Vietnam. Any SS recoveries that are slightly greater than 100% are expected due to normal statistical fluctuations in the outcomes of various analytical steps.

We calculated coefficient of variation (CV) values for each analyte found. The overall mean CV (±1 sd) values averaged over all samples were very low (ie, the estimates can be considered precise): for analyte level results at ≥1000 µg/g, CV = 2.7% (±2.7%); for results at <1,000 µg/g but ≥100 µg/g, CV = 3.1% (±5.7%); and for results at <100 µg/g, CV = 5.3% (±6.9%).

### Reported Values

We calculated the level of other flavor chemicals (OFCs) by subtracting the chemical levels of menthol, five clove compounds (eg, eugenol), and two common cigarette filter plasticizers (triacetin and triethyl citrate) from the total value for all 180 analytes. We calculated and report averages based on duplicate extractions. Each value was a confirmed GC/MS result based on authentic chemical standards, with the final internal standard-corrected quantitation value based on calibration standards. Values for nicotine are not reported because the extraction method was not optimized for alkaloids. Flavor chemical values near 0.001 mg per filter, rod, and stick (filter plus rod) are viewed as *estimated* as they were generally below the analytical calibration range. The reporting limit for this work was set at 0.001 mg/stick; lesser values are given as “not determined” (ND).

## Results

**Figure 1.**
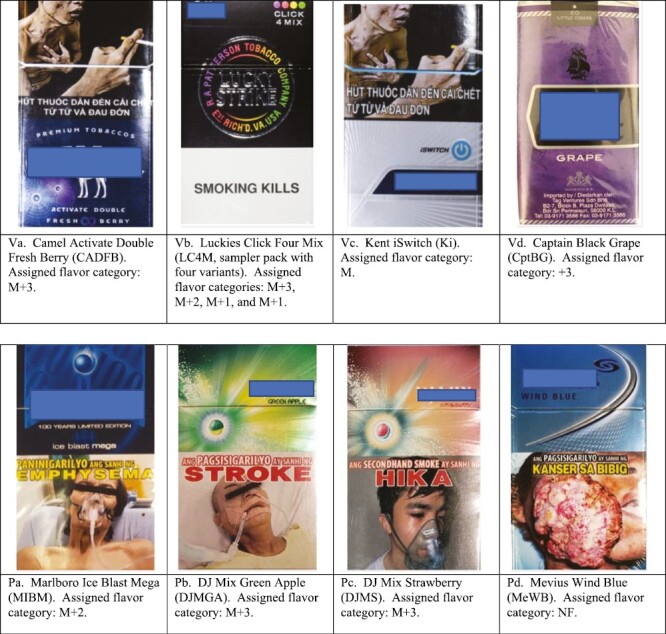
Pack photos of four of the 31 cigarette packs purchased in Vietnam (Va-Vd) and of four of the 19 cigarette packs purchased in the Philippines (Pa-Pd) in 2020. (Photos of all the Vietnam packs are provided in Supplementary Figures S-V1.a-S-V35.a and photos of all the Philippines packs are provided in Supplementary Figures S-P1.a-S-P19.a..

All the cigarettes in this convenience sample contained filters. Table 1 (Vietnam) and Table 2 (Philippines) indicate the brand names and the number of flavor capsules in the filter, along with levels of three categories of flavor analytes: clove, menthol and other flavor chemicals (OFCs). Of the 35 cigarette variants from Vietnam, 11 contained one crushable capsule and 9 contained two flavor capsules. The use of two capsules with different flavor mixes offers four options: crush neither, crush one, crush the other, and crush both. Of the 19 cigarette brand variants from the Philippines, 10 contained one crushable capsule and two contained a flavor thread (ie, a thread included in the filter for added flavor). Exemplar photos of packs are provided in Figure 1; photos of all cigarettes packs, including photos showing the opened filter and any capsules or threads, are provided at globaltobaccocontrol.org/flavorunderfire—Supplementary Figures S-V1.a/b–S-V36 (Vietnam) and Supplementary Figures S-P1.a/b–S-P20 (Philippines).

**Table 1.** Vietnam: Analytical results and flavor class designations for 35 cigarette variants from 32 packs (including 1 sampler pack with four subvariants) purchased in 2020.

Cigarette variant name	Abbreviation	Filtered (f) or non-filtered (nf), 1c = 1 flavor capsule, 2c = 2 flavor capsules, 1s = 1 flavor thread, carbon = carbon in filter	Flavor category^a^	Clv5 = Σ (5 clove compounds)^b^ (mg/stick)	Menthol (#90) (mg/stick)	Other flavor chemicals (OFCs)^c^ (mg/stick)	Total fruit-flavor chemicals (TFFCs)^d^ (mg/stick)
*Rank ordered based on menthol level*							
Marlboro Dry Menthol	MDM	f, 1c	M+5	0.02	16.6	4.9	4.6
Marlboro Double Burst	MDB	f, 2c	M+2	0.03	16.6	1.6	0.17
Lucky Strike Double Click Yellow	LSDCY	f, 2c	M+4	0.07	15.7	3.6	0.70
Marlboro Purple Burst	MPB	f, 2c	M+1	0.00	15.4	0.5	0.33
Lucky Strike Double Click Violet	LSDCV	f, 2c	M+3	0.07	15.0	2.7	0.59
Luckies Click 4 Mix - Sampler/Green	LC4M-S/Green	f, 1c	M+3	0.07	13.1	2.2	0.19
Luckies Click 4 Mix - Sampler/Red	LC4M-S/Red	f, 1c	M+2	0.01	11.9	1.2	0.81
Marlboro Ice Blast	MIB	f, 1c	M+1	0.02	10.7	0.89	0.03
Blue Ice Double Blast	BIDB	f, 2c	M+1	0.01	10.0	0.87	0.35
Marlboro Splash Mega Purple	MSMP	f, 1c	M+2	0.00	8.1	1.9	1.7
Luckies Click 4 Mix - Sampler/Purple	LC4M-S/Purple	f, 1c	M+1	0.00	7.8	0.47	0.43
Mond Variance Blueberry Menthol	MoVBM	f, 2c	M+1	0.01	7.3	0.65	0.32
Camel Activate Double Fresh Berry	CADFB	f, 2c	M+3	0.01	6.0	2.6	0.78
Mond Variance Applemint Menthol	MoVAMM	f, 2c	M+2	0.01	6.0	1.0	0.50
Oris Pulse Menthol Orange	OPMO	f, 1c	M+2	0.01	5.5	1.3	0.62
Kent iSwitch	Ki	f, 1c, carbon	M	0.00	5.2	0.03	0.01
Zest Marula	ZM	f, 1c	M+2	0.01	5.0	1.4	0.77
Raison Ice Coffee	RIC	f, 1c, carbon	M+7	0.01	5.0	6.2	5.0
Luckies Click 4 Mix - Sampler/Yellow	LC4M-S/Yellow	f, 1c	M+1	0.00	4.3	0.70	0.31
Oris Pulse Super Slim Double Mix	OPSSDM	f, 2c	M+3/clove	0.51	3.6	2.5	2.5
*Rank ordered based on OFCs level*							
Captain Black Cherise	CptBC	f	+4	0.00	0.02	3.2	2.3
Captain Black Grape	CptBG	f	+3	0.00	0.02	2.5	2.1
Zouk	Zouk	f	+2	0.00	0.03	1.1	0.17
Canyon Vanilla	CanV	f	+1	0.00	0.03	0.97	0.17
Black Devil Café	BDC	f	+1	0.00	0.03	0.96	0.09
Mond Strawberry Superslims	MoSSS	f	+1	0.00	0.00	0.69	0.69
Mond Superslims Cherry	MoSSC	f	+1	0.00	0.00	0.65	0.63
Richmond Coffee Superslim	RCofSS	f	+1	0.00	0.00	0.56	0.45
Chapman No.3 Coffee	ChN3Cof	f	NF	0.00	0.00	0.28	0.23
Richmond Cherry Superslim	RChSS	f	NF	0.00	0.00	0.23	0.22
Chapman No.3 Cherry	ChN3Ch	f	NF	0.00	0.01	0.22	0.21
Captain Black Dark Crema	CptBDCr	f	NF	0.00	0.01	0.13	0.01
Camel Caster	CC	f, carbon	NF	0.00	0.00	0.08	0.08
Camel Filters	CF	f, carbon	NF	0.00	0.00	0.01	0.01
Kent Nanotek 2.0	KN2	f, carbon	NF	0.00	0.01	0.01	0.00

^a^M = menthol at ≥ 1 mg/stick; for OFCs, “+1” = OFCs at 0.3 mg/stick ≤ OFCs ≤ 1 mg/stick, or “+2” = OFCs at 1 mg/stick < OFCs ≤ 2 mg/stick, etc.

^b^Clv5 = sum for compounds #129, #133, #138, #148, #159 (eugenol, eugenol methyl ether, β-caryophyllene, α-caryophyllene (α-humulene), and acetyl eugenol (eugenol acetate).

^c^OFCs = other flavor chemicals = total for the 180 target flavor chemical analytes minus the sum for (Clv_5_ + menthol + triacetin + triethyl citrate).

^d^TFFCs = total fruit flavor chemicals; the 105 chemicals considered here to be in the “fruit” group are identified in Supplementary Table S1.

**Table 2.** The Philippines: Analytical results and flavor class designations for 19 cigarette variants from 19 packs purchased in 2020.

Cigarette variant name	Abbreviation	Filtered (f) or non-filtered (nf), 1c = 1 flavor capsule, 2c = 2 flavor capsules, 1s = 1 flavor thread, carbon = carbon in filter	Flavor category ^a^	Clv5 = Σ (5 clove compounds) ^b^ (mg/stick)	Menthol (#90) (mg/stick)	Other flavor chemicals (OFCs) ^c^ (mg/stick)	Total fruit- flavor chemicals (TFFCs) ^d^ (mg/stick)
*Rank ordered based on menthol level*							
Marlboro Ice Blast Mega	MIBM	f, 1c	M+2	0.03	18.4	1.5	0.03
Bohem Mojito Double	BoMD	f, 2c	M+2	0.01	12.2	1.4	0.73
Winston Intense Mint	WIM	f, 1s	M	0.00	9.8	0.03	0.01
DJ Mix Green Apple	DJMGA	f, 1c	M+3	0.01	9.7	2.2	1.5
Winston Extreme Mint	WEM	f, 1s	M	0.00	9.7	0.02	0.01
DJ Mix Blueberry	DJMB	f, 1c	M+1	0.00	9.7	0.89	0.69
Winston Purple Mint	WPM	f, 1c	M+1	0.00	9.5	0.80	0.68
Marlboro Fusion Purple	MFP	f, 1c	M+1	0.00	9.5	0.33	0.26
Mevius Option Duo	MeOD	f, 1c	M+2	0.00	8.8	1.1	0.94
Marlboro Black Menthol	MBM	f	M	0.00	8.6	0.04	0.01
DJ Mix Strawberry	DJMS	f, 1c	M+1	0.00	7.0	0.44	0.40
Bohem Cigar Slim-Fit Brown	BoCSFBr	f, 1c	M+2	0.01	4.7	1.3	0.90
Esse Pop	EP	f, 1c	M+1	0.01	4.4	0.56	0.13
Dunhill Menthol	DuM	f	M	0.00	4.0	0.03	0.01
Rank ordered based on OFCs level							
Winston Caster with LSS	WCLSS	f, carbon	NF	0.00	0.00	0.09	0.08
Camel White	CW	f	NF	0.00	0.01	0.02	0.02
Mevius Sky Blue	MeSB	f, carbon	NF	0.00	0.00	0.02	0.02
Marlboro Premium Black	MPB	f	NF	0.00	0.00	0.01	0.01
Mevius Wind Blue	MeWB	f, carbon	NF	0.00	0.00	0.01	0.01

^a^M = menthol at ≥ 1 mg/stick; for OFCs, “+1” = OFCs at 0.3 mg/stick ≤ OFCs ≤ 1 mg/stick, or “+2” = OFCs at 1 mg/stick < OFCs ≤ 2 mg/stick, etc.

^b^Clv5 = sum for compounds #129, #133, #138, #148, #159 (eugenol, eugenol methyl ether, β-caryophyllene, α-caryophyllene (α-humulene), and acetyl eugenol (eugenol acetate).

^c^OFCs = other flavor chemicals = total for the 180 target flavor chemical analytes minus the sum for (Clv_5_ + menthol + triacetin + triethyl citrate).

^d^TFFCs = total fruit flavor chemicals; the 105 chemicals considered here to be in the “fruit” group are identified in Supplementary Table S1.

The detailed analytical results for the 180 analytes are provided at globaltobaccocontrol.org/flavorunderfire as Supplementary Table 1 (Vietnam) and Supplementary Table 2 (Philippines), including percentages of each analyte found on the filters.

Supplementary Figure 1 (Vietnam) and Supplementary Figure 2 (Philippines) contain flavor chemical “heat maps” for all analytes detected at least once at ≥0.001 mg/stick for either country.

Five flavor options groupings were found among brand variants purchased in Vietnam: menthol + OFCs (*n* = 15), OFCs only (*n* = 8), nonflavored (*n* = 7), menthol + OFCs with a clove flavorant (*n* = 1) and menthol only (*n* = 1). Two of the three cigarillos were classified as OFCs only, and the third was nominally nonflavored. Three flavor groupings were found among brand variants purchased in the Philippines: menthol + OFCs (*n* = 10), nonflavored (*n* = 5), and menthol only (*n* = 4).

Among the 180 compounds analyzed, menthol was present at the highest levels across brand variants. For the products purchased in Vietnam, the highest menthol level was 16.6 mg/stick (Marlboro Dry Menthol and Marlboro Double Burst variants). For the products purchased in the Philippines, the highest menthol level was 18.4 mg/stick (Marlboro Ice Blast Mega variant).

Eugenol, the major flavor chemical in clove, was not present in any brand variant examined except one brand purchased in Vietnam (Oris Pulse Super Slim Double Mix). For this brand, the eugenol level was 0.51 mg/stick; a trace level (~0.001 mg/stick) of β-caryophyllene (another clove-related chemical) was also present. The percent-on-filter value (100%) suggests that eugenol may have been present in at least one of the two flavor capsules in the filter.

Supplementary Figure 3 is a mg/stick stacked bar plot of menthol, clove, and OFC values for the brand variants purchased in Vietnam. Among those brand variants with flavor chemicals, menthol was the most heavily used flavor additive. When menthol was present, OFCs were also present (except one menthol-only brand variant) and the level of menthol generally exceeded the OFC level. For example, the Luckies Click 4 Mix sampler pack purchased in Vietnam, the four versions (green, red, purple, and yellow) contained different levels of menthol (13.1, 11.9, 7.8, and 4.3 mg/stick, respectively). The OFC values were also different (2.2, 1.2, 0.47, and 0.70 mg/stick, respectively) and the dominant OFC varied as follows: (1) green, *p*-menthone (#85; minty); (2) red, piperonal (#132; cherry, vanilla)/γ-nonalactone (#139, coconut)/γ-decalactone (#158, fruity, peach, apricot); (3) purple, isoamyl acetate (#20; sweet, fruity, banana); and (4) yellow, limonene (#46; herbal citrus)/linalool (#68; citrus, orange, lemon, floral). There were four brand variants from the Vietnam sample that contained OFCs-only and did not contain menthol.

Supplementary Figure 4 is a stacked bar plot of the menthol, clove, and OFC values for the brand variants purchased in the Philippines. Among those brand variants with flavor chemicals, menthol was also the most heavily used flavor additive and there were four menthol-only brand variants in this sample. When menthol was present, its value always exceeded the value for the OFCs group. There were no OFCs-only brand variants in the Philippines sample. Variants with threads had as high menthol values as variants with capsules. Two variants in one of the M groupings had neither a capsule nor thread.

## Discussion

We found that most cigarette brand variants in our sample purchased in Vietnam and the Philippines contained menthol and OFCs. Among the 180 analytes tested, menthol was present at the highest amount across brand variants in both countries. Menthol makes cigarettes more palatable and can suppress respiratory symptoms^19^; the tobacco industry intentionally manipulates the level of menthol in cigarettes brand variants to appeal to different consumers,^20^ and individuals who regularly smoke menthol may prefer variants with higher menthol levels.^21^ Furthermore, those who smoke menthol have a lower likelihood of quitting despite making more quit attempts.^22–24^ Our findings suggest that brand variants for sale in these two countries include a range of menthol values that could appeal to consumers across this spectrum of users. Interestingly, the range of menthol values for these cigarettes purchased in 2020 was higher than that of our convenience sample of packs purchased in 2017 from Mexico.^16^ As such, any flavored tobacco ban considered in Vietnam or the Philippines must include menthol at a minimum to reduce the public health burden of this flavor additive.

However, our chemical analysis also reveals the presence of OFCs in brand variants purchased in both countries, either alone or, more commonly, in combination with menthol. Oftentimes, OFCs and menthol were found in flavor capsules or flavor threads. Two of the three cigarillos in our sample had flavor chemicals; flavored cigarillos were introduced in high-income countries when these countries implemented flavored cigarette bans and thus this is an area to monitor in low- and middle-income countries.

These findings highlight that in Vietnam and the Philippines menthol and flavors were readily available in cigarettes, some of which contained flavor “technologies” such as flavor capsules and flavor threads. Flavor capsules and threads are being used to appeal to new consumers.^25^ Evidence indicates that flavor chemicals, including fruit flavors, menthol and clove, and flavor capsule cigarettes, are appealing to young people.^26,27^ Filipino young adults even liken flavor capsule cigarettes to candy.^28^ The present study indicates that flavor chemicals and flavor delivery technology are readily available for sale in Vietnam and the Philippines, suggesting a comprehensive flavored tobacco ban that includes all flavors present in any component part of a cigarette or tobacco product is required in both countries.

This study is the first to analyze the chemical composition of cigarette brand variants available in Vietnam and the Philippines. The major strength of this analysis is our ability to document flavored cigarette brand variants available for sale in both countries. However, given this was a convenience sample the analysis cannot be generalized to all flavored cigarette variants available across Vietnam and the Philippines.

While rates of smoking in Vietnam and the Philippines have decreased since 2010, both countries have fallen behind in their respective goals of a 30% reduction in smoking by 2025. A comprehensive flavor ban that includes all flavors present in all cigarette components, including flavor capsules and threads, is one pathway to reduce cigarette sales and promote smoking cessation in these two countries and the Western Pacific region.^29–31^ Findings from the present study can inform efforts to develop and implement flavored tobacco policy initiatives.

## Supplementary Material

A Contributorship Form detailing each author’s specific involvement with this content, as well as any supplementary data, are available online at https://academic.oup.com/ntr.

ntad146_suppl_Supplementary_Figure_S1

ntad146_suppl_Supplementary_Figure_S2

ntad146_suppl_Supplementary_Figure_S3

ntad146_suppl_Supplementary_Figure_S4

## Data Availability

The data are available in the supplementary tables and figures; additional data requests should be sent to the corresponding author.
