# Supplementary figures and images for: Menthol and Other Flavor Chemicals in Cigarettes from Vietnam and the Philippines
Source: Nicotine Tob Res. 2023 Aug 14;26(3):385–91. doi: 10.1093/ntr/ntad146 (PMC10882432; doi:10.1093/ntr/ntad146)

Supplementary figure 1.

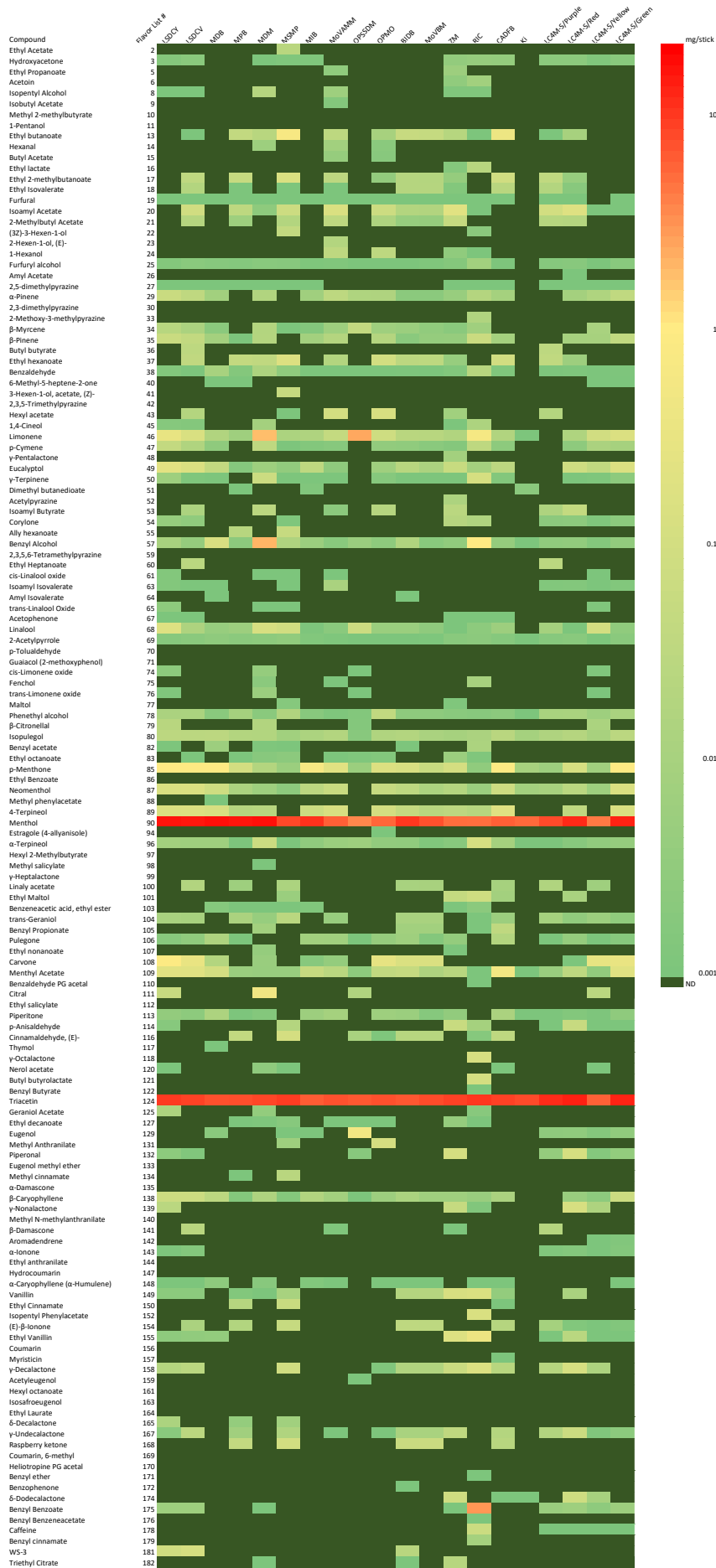

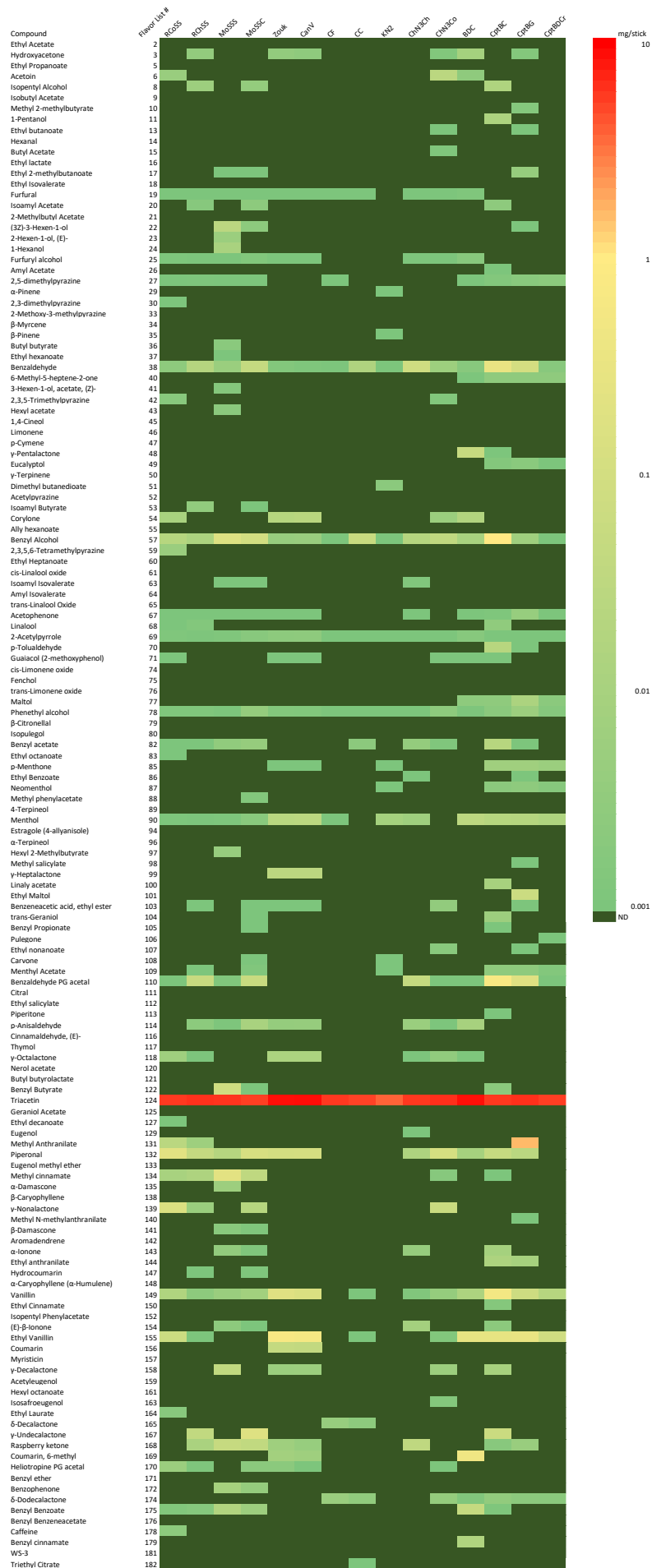

Supplement: ntad146_suppl_Supplementary_Figure_S1 [file ntad146_suppl_supplementary_figure_s1.pdf]

Supplementary figure 2.

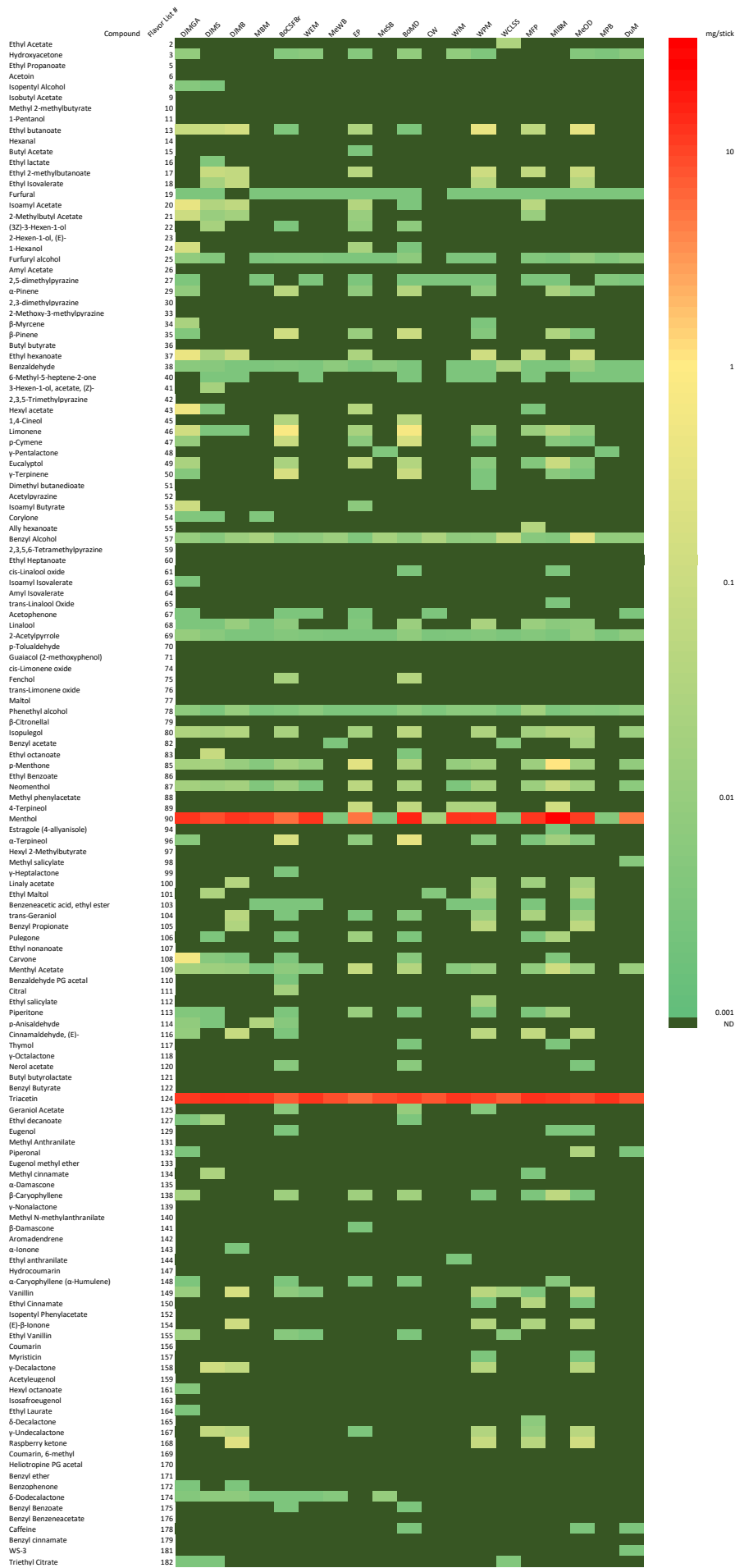

Supplement: ntad146_suppl_Supplementary_Figure_S2 [file ntad146_suppl_supplementary_figure_s2.pdf]
