# Supplementary material for: Menthol and Other Flavor Chemicals in Cigarettes from Vietnam and the Philippines
Source: Nicotine Tob Res. 2023 Aug 14;26(3):385–91. doi: 10.1093/ntr/ntad146 (PMC10882432; doi:10.1093/ntr/ntad146)

Supplementary Figure 3. Stacked bar plot for 35 cigarette variants (in 32 brand packs with one being a "sampler" pack holding four different variants) purchased in Vietnam in 2020 giving mg/stick values for menthol, Clv5, and OFCs, with OFCs = mg/stick sum over 180 target analyte flavor chemicals minus the sum for (menthol + Clv5 + triacetin + triethyl citrate).

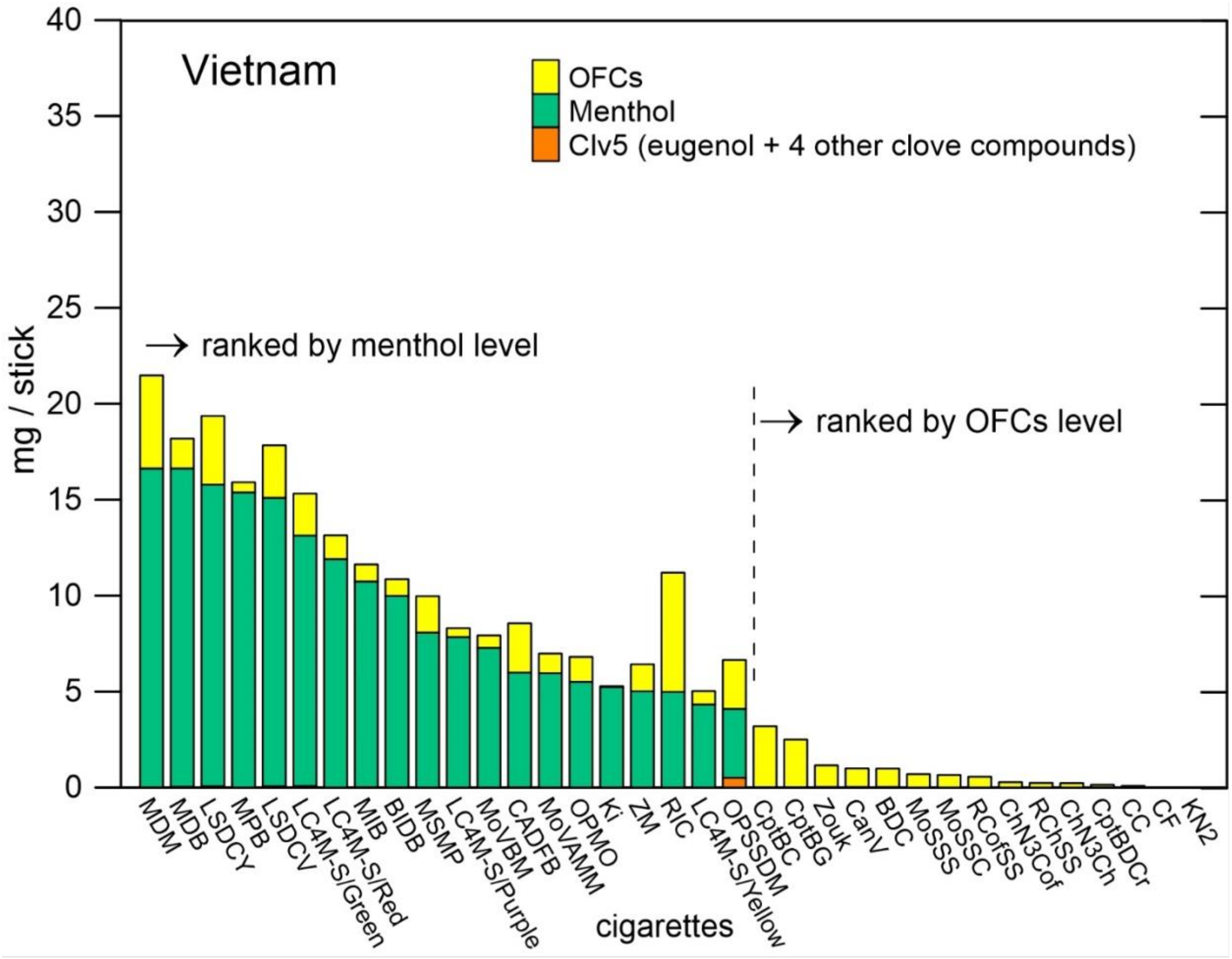

Supplement: ntad146_suppl_Supplementary_Figure_S3 [file ntad146_suppl_supplementary_figure_s3.pdf]
